# Frequency and risk factors for hydroxychloroquine retinopathy among patients with systemic lupus erythematosus

**DOI:** 10.1186/s43162-021-00047-y

**Published:** 2021-06-08

**Authors:** Mohammed Salah Eldin Abdelbaky, Tarek Ahmad El Mamoun, Fatma Ibrahim Mabrouk, Rasha Mohamad Hassan

**Affiliations:** 1 Department of Medicine Division of Rheumatology, Ain Shams University, Cairo, Egypt; 2 Department of Ophthalmology, Ain Shams University, Cairo, Egypt

**Keywords:** SLE, Hydroxychloroquine retinopathy, Frequency, Risk factors, Fundus autofluorescent

## Abstract

**Background:**

Hydroxychloroquine (HCQ) is an antimalarial drug, recently used in COVID-19 treatment. Also it is considered over many years the cornerstone in treating systemic lupus erythematosus (SLE) in adults and children. The incidence of retinal affection and retinal toxicity from hydroxychloroquine is rare, but even after the HCQ is stopped, loss of vision may not be reversible and may continue to progress. Fundus autofluorescence (FAF) is one of the screening methods recommended by AAO used for the diagnosis of hydroxychloroquine retinopathy. Our aim is to detect early HCQ-induced retinopathy among SLE patients and the risk factors for its development by using fundus autofluorescence.

**Results:**

In the present study, 11.3% of the studied patients had significant visual field changes upon testing. Of those, 6.3% had abnormal fundus autofluorescence. We found a significant statistical relation between hydroxychloroquine retinopathy and the duration and cumulative dose of hydroxychloroquine therapy (*p* value = 0.003) and decreased best-corrected visual acuity of both eyes (*p* value = 0.000). There was no relationship between HCQ retinopathy detected by fundus autofluorescence and daily dose of HCQ/kg, age, sex, and SLEDAI score.

**Conclusion:**

Frequency of SLE patients who had confirmed HCQ-induced retinopathy was 6.3%. Hydroxychloroquine could be safely used in all SLE patients regardless of age, sex, and SLE activity. Routine ophthalmological assessment is recommended for SLE patients who received HCQ especially for those who received HCQ longer than 7 years. Fundus autofluorescence is a modern objective tool which is specific for the early detection of HCQ retinopathy.

## Background

HCQ is used in treating SLE in adults and children. It is effective for the treatment of the specific skin lesions of cutaneous LE (CLE) [1], whether acute, subacute, or chronic. It is very effective for the amelioration of joint and constitutional symptoms and for the prevention of clinical relapse [30].

The incidence of retinal toxicity induced from hydroxychloroquine is rare, but even if the HCQ is stopped, loss of vision may not be reversible [18, 20] and may continue to be more advanced [16]. It is important that patients and doctors are aware of and observe this drug ocular side effect. And before starting treatment with hydroxychloroquine, a complete ophthalmic assessment should be done to detect any baseline maculopathy [30].

Patients are usually asymptomatic, and their visual acuity is preserved at the early stages. Actually, objective changes precede a patient’s complaint of vision loss. Rarely, when the investigation was done, the patient may be found to have minimal changes in night vision, diminished color vision, or a paracentral scotoma [14]. Paracentral scotoma which is experienced by the patient can worsen, and the patient often has a subjective complaint of reading difficulty that occurs before any changes are found on dilated fundus examination [15].

Two years ago, Royal College of Ophthalmologists (RCOphth) recommended that all patients received hydroxychloroquine for greater than 5 years should undergo 10-2 Humphrey visual field testing (using a white stimulus), followed by pupillary dilation and imaging with both spectral domain optical coherence tomography (SD-OCT) and/or wide field fundus autofluorescent (FAF) [35].

Fundus autofluorescence (FAF) is one of the objective screening methods for HCQ retinopathy [9]. A parafoveal increased autofluorescence signal is detected in the early stages of disease, with mottled decrease in parafoveal autofluorescence as Retinal Pigment Epithelium (RPE) degeneration becomes established in later stages of disease [9].

### Aim of the study

This study was designed to get frequency and risk factors for hydroxychloroquine retinopathy among patients with systemic lupus erythematouses using fundus autofluorescence.

## Methods

The current study included 80 systemic lupus erythematosus patients who were diagnosed according to Systemic Lupus International Collaborating Clinics (SLICC) classification criteria [24]. They were recruited from the outpatient rheumatology clinic and inpatients at the Department of Internal Medicine, Rheumatology Division. All patients have no visual symptoms; also, other causes of retinopathy (DM, HTN, drug induced as tamoxifen) were excluded from the study. The study was approved by the local research ethical committee. All participants gave written consent after explaining the nature of the study.

The study is a cross-sectional observational study. All patients were subjected to the following:

Clinical assessment: Full medical history was taken from each patient, including information about duration and dosage of HCQ intake. Rheumatological examination as well as assessment of disease activity using systemic lupus erythematosus disease activity index (SLEDAI) were done [2]*.*

Laboratory assessment is as follows: CBC, ESR, CRP, serum complement level (C3 and C4) by radial immunodiffusion assay, complete urine analysis, urinary protein creatinine ratio, anti-nuclear antibody (ANA) by immunofluorescent test, anti-dsDNA by ELISA, serum creatinine, blood urea nitrogen (BUN), and fasting blood glucose.

Ophthalmological assessment is as follows: fundus examination by an experienced ophthalmologist, best-corrected visual acuity by Landolt chart on 6 m and expressed in decimal, and the 10-2 Humphrey visual field analyzer. Visual field images of 80 patients were collected. Scotoma point locations were also recorded; abnormality was defined as partial or full ring scotoma mainly affecting the parafoveal region [6].

Fundus autofluorescence is as follows: it was done by an experienced opthalmologist who was blinded to the clinical and laboratory data, by using a scanning laser ophthalmoscopy VX-20 retinal camera (KOWA, JAPAN). The intensity of the FAF image depends on the concentration and amount of lipofuscin and to a lesser extent, other fluorophores. Lesions can be classified as hyper-autofluorescent, results from alterations in lipofuscin metabolism, and its accumulation in RPE cells; hypoautofluorescent arises from decreased amount of lipofuscin or PRE atrophy [9, 34].

**Fig. 1:**
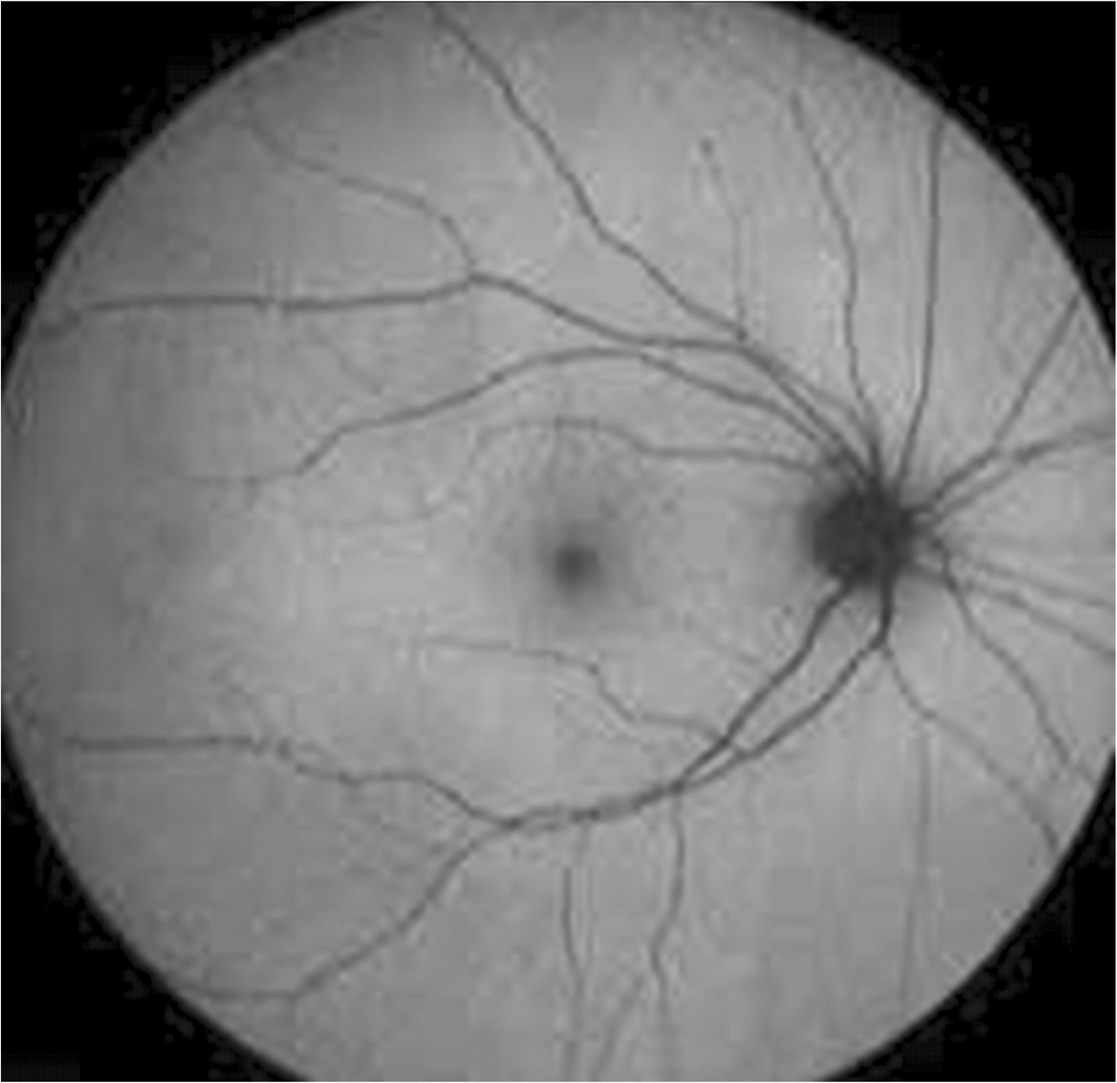
Normal fundus autoflourscent

**Fig. 2:**
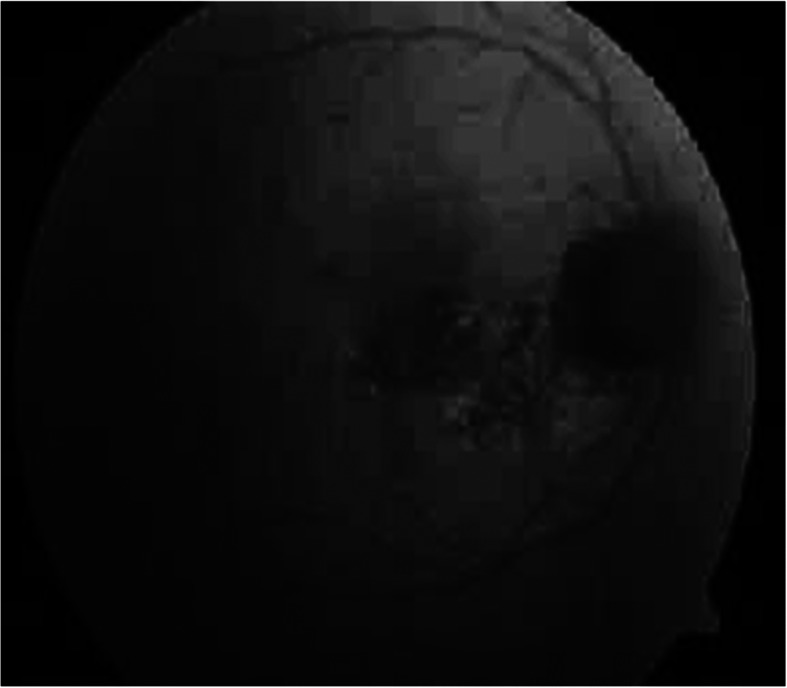
Retinal Pigmented Epithelium by fundus autoflourscent (RPE)

**Fig. 3:**
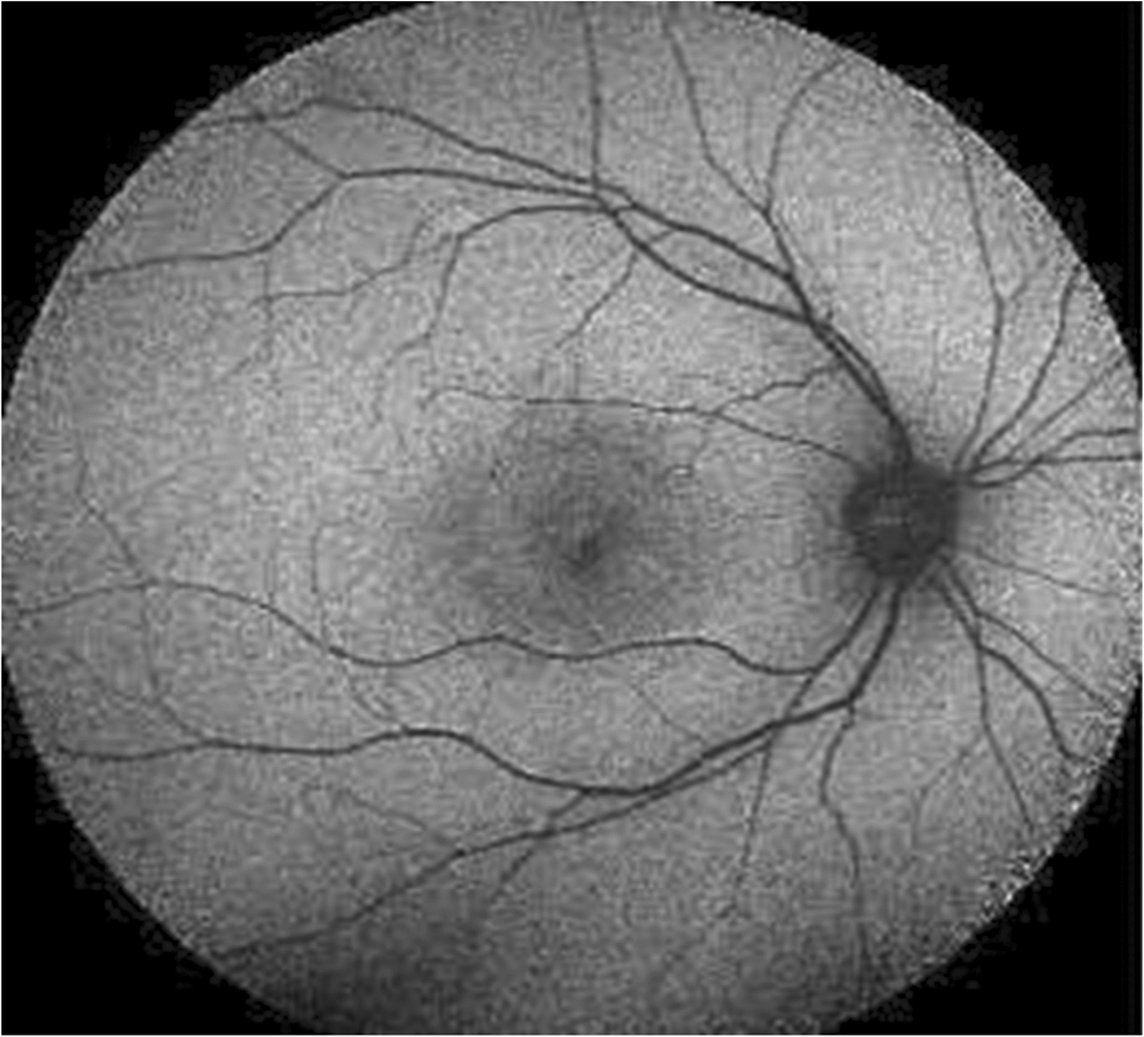
Bull’s eye by fundus autofluorescent

In HCQ-induced retinopathy, FAF images show early hyper-autofluorescent parafoveal ring corresponding to photoreceptor damage, which decreases in signal strength over time due to RPE atrophy (hypoautofluorescent). Advanced stages of the disease include bilateral bull’s-eye maculopathy. The area of hypoautofluorescent may spread to involve the central fovea and even the entire fundus [9] (Figs. 1, 2, and 3)

As regards the finding of the fundus autofluorescent, the patients will be divided into 2 groups: group 1, patients who have normal FAF; and group 2, who have abnormal FAF.

### Statistical analysis

The data were collected, tabulated, and statistically analyzed. Analysis of data was done by a personal computer using SPSS (Statistical Program for Social Science) under Windows version 17 as follows: description of quantitative variables as mean, standard deviation (SD), and range; description of qualitative variables as number (no) and percentage. Chi-square test was used to compare qualitative variables. Unpaired *t* test was used to compare two independent groups as regards quantitative variables. Spearman’s correlation co-efficient rank test was used to rank different variables against each other positively or inversely (*p* > 0.05 = insignificant, *p* ≤ 0.05 = significant, *p* ≤ 0.001 = highly significant).

Receiver operating characteristic curve (ROC) was used to assess the best cutoff point with its sensitivity, specificity, positive predictive value (PPV), negative predictive value (NPV), and area under curve (AUC).

### Ethical consideration

Approval of study conduction was obtained from the Ethical Review Committee at the Faculty of Medicine, the purpose of this study was explained to all participants, confidentiality was assured, and written consent was obtained from all participants**.**

## Results

Eighty SLE patients on hydroxychloroquine therapy were included. The clinical data are shown in Table 1. Ninety percent of studied patients were females and 10% were males; their mean ages were 31.24 ± 7.41 range from 18 to 50 years. Their mean daily HCQ dose/kg was 4.89 ± 1.01 ranges from 2.2 to 7.1 mg/kg.

**Table 1:** Clinical data of SLE patients at the time of enrollment

Clinical parameter	***N*** (%)	Mean	Range
**Age (Y)**		31.24 ± 7.41	18–50
**Sex (F/M)**	72 (90%)/8 (10%)		
**Hcq therapy**			
**Duration of HCQ therapy (years)**			0.3–15
_**Daily dose mg/kg**_		4.89± 1.01	2.2–7.1
**Cumulative dose (g)**		713.67 ± 498.75	36.5 ± 2190
**SLEDAI**			0–25
**BCVA**		0.85 ± 0.30	0.1–1/0.16–1
**ESR (mm/h)**			5–140
**ANA positive (IU/ml)**	75 (93.8%)		
**Ant-DNA positive (U/ml)**	49 (61.3%		
**Fundus**			
**Normal**	77 (96.2%)		
**Bull’s eye**	3 (3.8%)		
**Fundus autofluorescent**			
**Normal**	75 (93.8%)		
**Abnormal (bull’s eye/RPE)**	5 (6.3%)		
**Visual field**			
**Normal**	68 (85.0%)		
**Significant change**	9 (11.3%)		
**Non-reliable**	3 (33.8%)		

*SLEDAI* Systemic Lupus Erythematosus Disease Activity Index, *HCQ* hydroxychloroquine, *BCVA* best-corrected visual acuity, *ESR* erythrocyte sedimentation rate, *FAF* fundus autofluorescence, *ANA* antinuclear antibody, *Anti-DNA* anti-deoxyribonucleic acid, *RPE* retinal pigmented epithelium

The HCQ therapy duration ranges from 0.3 to 15 years. According to the SLEDAI score, 15% of the studied patients are in remission, 27.5% are having mild disease activity, 28.8% are having moderate activity, 22.5% are having high activity, and 6.2 % are having very high activity. Visual field tests were done for all patients taking HCQ. Significant changes in visual field tests were found in nine patients (11.3%). FAF confirmed hydroxychloroquine retinopathy in (6.3%) of those patients.

Comparison between patients who have normal FAF (group 1) and those who have abnormal FAF (group 2), there were statistically significant differences between patients who have normal FAF (group 1) and those who have abnormal FAF (group2) as regards the duration of hydroxychloroquine therapy (*p* = 0.003), cumulative dose of HCQ therapy (*p* = 0.000), and decreased the best-corrected visual acuity of both eyes (*p* = 0.000) (Table 2).

**Table 2:** Comparison between normal FAF group and abnormal FAF group

		Normal FAF	Abnormal FAF	Test value	***P*** value	Sig.
		No. = 75	No. = 5			
**Age**	**Mean**	31.15 **±**7.22	32.60 **±** 10.85	0.423	0.674	NS
**Sex**	**F/M**	68/7 (90/9%)	4/1 (80/20%)	0.593	0.441	NS
**Duration of HCQ therapy (years)**	**Median (IQR)** **Range**	5 (2–6) 0.3–15	10 (8–10) 8 – 15	− 3.017^c^	0.003	HS
**Daily dose (mg)/kg**	**Mean± sd** **Range**	4.86 **±** 1.01 2.2–7.1	5.28**±** 0.88 4.4–6.6	− 0.896^a^	0.373	NS
**Cumulative dose (g)**	**Mean± sd** **Range**	661.96 ± 461.26 36.5–2190	1489.20 ± 418.08 1168–2190	− 3.901	0.000	HS
**Fundus**	**Normal**	75 (100%)	2 (40.0%)	46.753^a^	0.000	HS
	**RPE**	0 (0%)	3 (60.0%)			
**BCVA RT**	**Mean ± SD**	0.88 ± 0.27	0.40 ± 0.37	3.702^b^	0.000	HS
	**Range**	0.1–1	0.1–1			
**BCVA LT**	**Mean ± SD**	0.93 ± 0.19	0.31 ± 0.17	6.873^b^	0.000	HS
	**Range**	0.16–1	0.16–0.5			

*P* value > 0.05: non-significant (NS); *P* value < 0.05: significant (S); *P* value < 0.01: highly significant (HS)

*SLEDAI* Systemic Lupus Erythematosus Disease Activity Index, *HCQ* hydroxychloroquine, *BCVA* best-corrected visual acuity, *FAF* fundus autofluorescence, *RPE* retinal pigmented epithelium

^a^Chi-square test

^b^Independent *t* test

^c^Mann-Whitney test

The mean weight-based daily dose of HCQ in group 1 was 4.86 ± 1.01, and in group 2 was 5.28 ± 0. 88. There was no significant statistical difference between both groups regarding the daily dose of hydroxychloroquine therapy.

The mean age in group 1 was 31.15 **±**7.22, and in group 2 was 32.60**±** 10.85. There was no significant statistical difference between both groups regarding age. Regarding the SLEDAI score, it was ranged from 0 to 25 in group 1 and ranged from 5 to 20 in group 2. There was no statistically significant difference between both groups regarding SLEDAI score.

**Fig. 4:**
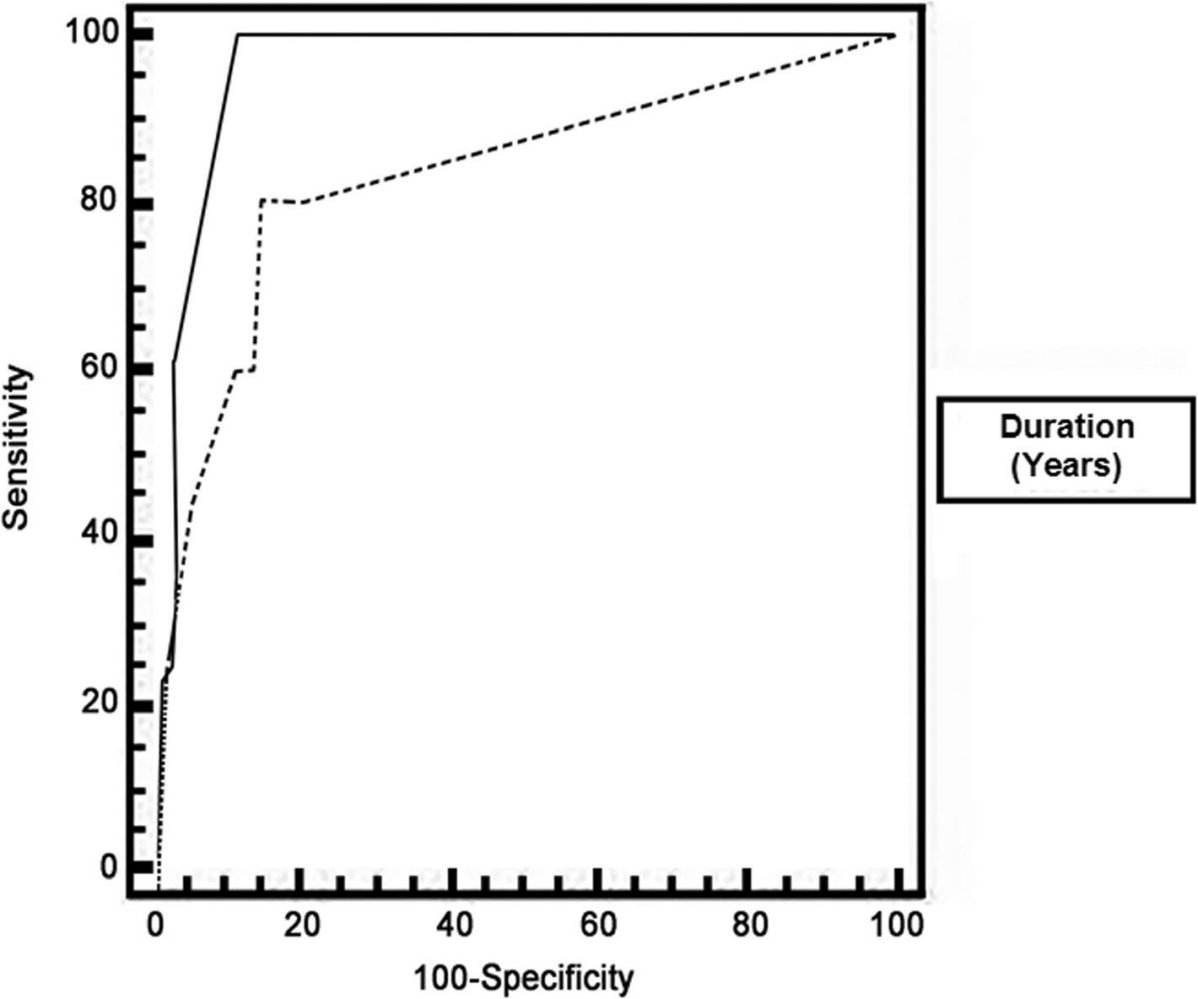
Receiver operating characteristic curve (ROC) for (HCQ Duration) as a predictor of abnormal FAF cases

**Fig. 5:**
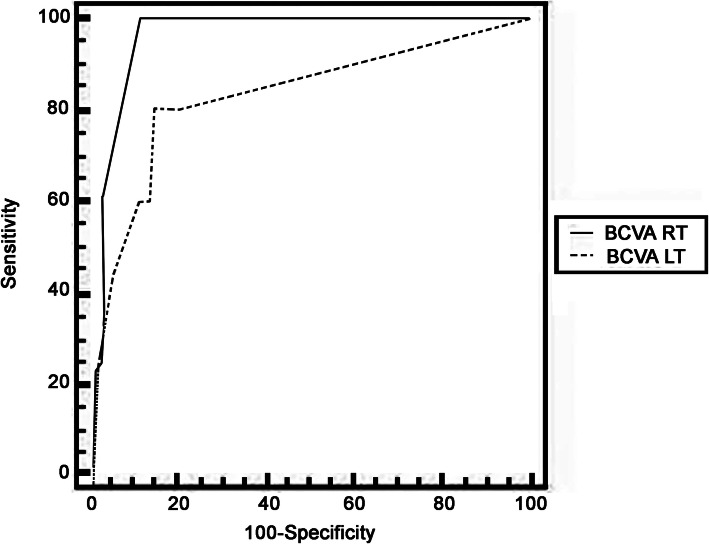
Receiver operating characteristic curve (ROC) for (BCVA) as a predictor of abnormal FAF cases

The sensitivity and specificity of FAF (the ability of FAF to detect the true positive and true negative HCQ retinopathy cases) in relation to the visual field test was 59% and 100% with accuracy of 94% (Tables 3 and 4). Predictors for abnormal FAF cases (by using calculating the receiver operating characteristic curves) were the duration of hydroxychloroquine therapy longer than 7 years and best-corrected visual acuity (BCVA) of both eyes ≤ 0.5 (Figs. 4, 5 and Table 5).

**Table 3:** Comparison between the 10–12 visual field and FAF

FAF Normality	Normal Visual field		Abnormal Visual field		Test value	***P*** value	Sig.
	No.	%	No.	%			
**Normal**	68	100.0%	4	44.4%	40.401	< 0.001	HS
**Abnormal**	0	0.0%	5	55.6%

**Table 4:** Diagnostic accuracy of FAF in prediction of visual field results

Variables	Accuracy	Sensitivity	Specificity	+PV	−PV
FAF	94.81%	55.6%	100.0%	100.0%	94.4%

**Table 5:** Receiver operating characteristic curve (ROC) for predictors of abnormal FAF cases

Variables	Cutoff point	AUC	Sensitivity	Specificity	+PV	−PV
**Duration of therapy (years)**	> 7	0.900	100.00	82.67	27.8	100.0
**BCVA RT**	≤ 0.5	0.832	80.00	85.33	26.7	98.5
**BCVA LT**	≤ 0.5	0.963	100.00	89.33	38.5	100.0

*BCVA* best-corrected visual acuity, *AUC* area under curve, *+PV* true positive value, *−PV* false positive value

The previous table shows that FAF can predict visual field results with a sensitivity of 55.6%, specificity of 100.0%, and accuracy of 94.81.

## Discussion

Over the last decade, clinicians have emphasized the role of hydroxychloroquine in SLE treatment [8, 27]. Not only its efficacy in preventing SLE flares and damage, but it has an important role against the development of diabetes mellitus, thrombotic events, and dyslipidemia in patients with SLE. This results in improving the survival in SLE patients [4].

A well-known complication in long-term HCQ intake and high cumulative dose therapy is the development of a HCQ-related maculopathy, carrying the possibility of occurrence of irreversible sight-threatening bull’s-eye maculopathy (BEM). The primary site of toxicity in HCQ retinopathy is the photoreceptor layer with secondary degeneration of the retinal pigmented epithelium (RPE) [26].

This study was designed to get frequency and risk factors for hydroxychloroquine retinopathy among patients with systemic lupus erythematous.

It included 72 females (90%) and 8 males (10%), whose ages ranged from 18 to 50 years with mean age of 31.24 years. The higher frequency of SLE among women has resulted from the estrogen hormonal effect [5]. SLE can occur at any age; it is most common in childbearing age although it has been reported in both extremes of life [10].

In the current study, the frequency of confirmed hydroxychloroquine retinopathy by using 10–12 Humphury visual field and FAF was 6.3%. This was higher than Tangtavorn et al. [29] and Cabral et al. [3] who found the prevalence of toxic retinopathy caused by antimalarial drugs were 3.2% and 4.15%, respectively. However, it is less than Palma Sanchez et al. [25] who demonstrated in a retrospective study an overall rate of retinal toxicity (13.5%)*.* The previous studies used another method (SD-OCT and electoretinogram (ERG) plus 10–12 visual field testing) for detection of hydroxychloroquine retinopathy in different rheumatological diseases (SLE and RA).

Using the visual field testing showed significant changes in another 4 patients who were considered as having possible hydroxychloroquine retinopathy by the Royal College of Ophthalmology (RCOphth) definition of the hydroxychloroquine retinopathy [35]. This proved the high sensitivity of the 10–12 Humphery visual field test. Melles and Marmor [17] reported that visual field testing is considered the most sensitive tool for detecting the earliest stages of retinopathy, although its results are subjective and affected variably by patient responses.

Between the studied risk factors, the hydroxychloroquine retinopathy depends mainly on the duration of hydroxychloroquine therapy. This is in agreement with a number of studies related to the risk of retinopathy to increase duration of HCQ treatment (Palma Sanchez et al. [25]; Wolfe and Marmor [31]). Also, Petri et al. [23] found that there was a 1% risk of retinopathy in the first 5 years of HCQ treatment, 1.8% from 6 to 10 years, and 3.3% from 11 to 15 years, so the risk increases after 16 years of use, even after 20 years.

It is known that hydroxychloroquine takes about 1 month and needs about 6 months to achieve full elimination from the body. Hydroxychloroquine toxicity is caused by the accumulation of the drug in the outer retinal layer leading to macular edema and or bilateral granular depigmentation of the RPE in the macula with continued exposure to the drug. This can progress to an atrophic bull’s eye with concentric rings of hypopigmentation and hyperpigmentation surrounding the fovea. These changes can progress to include other areas of the fundus causing widespread atrophy [19, 32].

Moreover, the Royal College of Ophthalmologists (RCOphth) 2009 guidelines preferred ophthalmological screening for patients who have received HCQ therapy for more than 5 years. This became evidence since 2011.

Also, we found that the cumulative dose of the hydroxychloroquine therapy was significantly related to the hydroxychloroquine retinopathy that was in agreement with Petri et al. [23] and Lenfant et al. [11]. Previously, the cumulative dose was described as one of the risk factors of the hydroxychloroquine retinopathy [14, 17]. In 2016, Marmor et al. reported that the patients using a recommended dose had significant risk after decades of use. But this risk is mainly dependent on the duration of use relative to the daily dose based on body weight.

In this study, weight-based daily dose was not a risk factor for HCQ retinopathy. However, the previous studies emphasized that the daily dose of more than 5 mg/kg is one of the most important risk factors for HCQ retinopathy [7, 11, 17]. The explanation might be that most of the studied patients’ weights were above 70 kg and most of them received 400 mg/day or less, also the low socioeconomic level of our patients might make them non-compliant to the treatment.

In 2011, American Academy of Ophthalmology (AAO) guidelines (screening for HCQ retinopathy) advised weight-based HCQ dosing of 6.5 mg/kg/day with a maximum dose of 400 mg/day (exceptions were obese and short stature patients, dosage based on ideal body weight was used). Finally, the latest 2016 AAO guidelines and 2018 RCOphth guidelines returned to use ABW and recommended < 5.0 mg/kg of ABW [35].

Regarding age and sex, there was no significant relation between hydroxychloroquine retinopathy and the age and sex of examined patients. This agrees with similar results found by Yaylali et al. [33] and Levy et al. [12] while Petri et al. [23] and Ding et al. [7] found that increased age above 60 years is considered a risk factor for HCQ retinopathy. We did not find these results as most of our patients are females (90%) and their ages ranged from 18 to 50 years.

Also, we found no significant relation between hydroxychloroquine retinopathy and SLE activity by SLEDAI score and the immunological markers. This was consistent with the recent study done by Lenfant et al. [11]. HCQ is a very important drug for SLE treatment; up till now, it cannot be proved that the disease activity has a role in the pathogenesis of HCQ retinopathy.

By comparing the routine fundus examination and fundus autofluorescence, the latter was better for detecting early hydroxychloroquine retinopathy. This was already reported in many previous studies [22, 28]. They reported that patients on HCQ therapy and had no clinical retinopathy, fundus autofluorescence is helpful to early detect and monitor for retinal affection in patients taking HCQ.

FAF was found as a specific method but not sensitive for early detection of the Hcq retinopathy in relation to the visual field testing (the sensitivity 59%, the specificity 100%). This is in agreement with Marmor [15] and Pandya et al. [21] who reported that FAF is not recommended as a primary screening tool. Instead, it is recommended by the AAO guidelines that FAF must be used with other imaging tools [13].

In this study, the duration of HCQ therapy of more than 7 years was a predictor for HCQ retinopathy among our patients by using the fundus autofluorescence. This is in agreement with Wolfe and Marmor [31] and also Mavrikakis et al. [16] in a prospective cohort study to assess retinal toxicity among patients who received the recommended dose. They had no retinal toxicity during a follow-up period of 6 years. In 2016, the American Academy of Ophthalmology recommended screening for those who received HCQ more than 5 years [13]. We hypothesized that non-compliance of our patients may prolong the duration needed for the occurrence of the retinopathy.

## Conclusion

Frequency of SLE patients who had confirmed HCQ-induced retinopathy was 6.3%. Hydroxychloroquine could be safely used in all SLE patients regardless of age, sex, and SLE activity, and routine ophthalmological assessment is recommended for SLE patients who received HCQ especially for those who received HCQ longer than 7 years. Fundus autofluorescence is a modern objective tool which is specific for early detection of HCQ retinopathy.

Limitations of our study should be pointed out. One of the limitations of our study is that like most others, it was a cross-sectional study. Another limitation is that all of the included patients come from a low-socioeconomic level that may affect compliance, follow-up, and SLE treatment strategy. In addition, spectral domain optical coherence tomography and multifocal electroretinogram (mfERG) were not available for full assessment for the presence of any retinopathy related to HCQ treatment.

## Data Availability

The data that support the finding of this study are available from Rasha Mohamad Hassan (corresponding author).

## Abbreviations

AAO: American Academy of Ophthalmology
ABW: Actual body weight
BCVA: Best-corrected visual acuity
DM: Diabetes mellitus
HCQ: Hydroxychloroquine
HTN: Hypertension
RCOphth: Royal College of Ophthalmologists
SD-OCT: Spectral domain optical coherence tomography
SLE: Systemic lupus erythematosus
SLEDAI: Systemic Lupus Erythematosus Disease Activity Index
SLICC: Systemic Lupus International Collaborating Clinics
